# Assessment of Serum Ferritin Levels in Female Patients With Telogen Effluvium

**DOI:** 10.7759/cureus.100249

**Published:** 2025-12-28

**Authors:** Nishantan Thamotharan, Manu V Harikumar, Murugan Sundaram, Adikrishnan Swaminathan, Sudha Rangarajan

**Affiliations:** 1 Dermatology, Sri Ramachandra Institute of Higher Education and Research, Chennai, IND

**Keywords:** alopecia, iron deficiency, serum ferritin, stress, telogen effluvium

## Abstract

Background

Telogen effluvium (TE) is a non-scarring form of diffuse hair loss characterized by premature transition of hair follicles into the telogen phase, resulting in excessive shedding. Iron deficiency, reflected by low serum ferritin levels, is considered a potential contributor to TE in women.

Aim

The aim of this study was to evaluate serum ferritin levels in female patients with TE and compare them with controls.

Methods

A cross-sectional comparative study was conducted involving 100 female participants - 50 diagnosed with TE and 50 healthy controls. Serum ferritin and hemoglobin levels were measured in both groups. Demographic, lifestyle, hormonal, and clinical variables were recorded to account for potential confounders. Data analysis was performed using SPSS version 20.0 (IBM Inc., Armonk, New York), employing independent t-tests for continuous variables and Chi-squared tests for categorical variables.

Results

Mean serum ferritin levels were significantly lower in TE cases (24.30 ± 11.13 ng/mL) compared to controls (44.78 ± 19.89 ng/mL; p<0.001). Reduced ferritin levels (<15 ng/mL) were observed in 14 (28%) TE cases, while none of the controls exhibited such deficiencies. Additionally, 31 (62%) TE cases demonstrated significant hair shedding, with a positive hair pull test in the same proportion.

Conclusion

Low serum ferritin levels are significantly associated with telogen effluvium in women. Serum ferritin may serve as a useful biomarker for identifying iron deficiency in patients presenting with diffuse hair loss.

## Introduction

Hair is a keratin-based filament rich in sulfur-containing amino acids, undergoing a cyclical pattern of growth and renewal. The hair follicle cycle comprises four distinct phases: anagen (active growth), catagen (regression), telogen (resting), and exogen (shedding) [1,2]. Telogen effluvium (TE) is a common, non-scarring alopecia characterized by premature termination of the anagen phase and a subsequent increase in telogen-phase hairs, leading to diffuse hair shedding [3].

The term "alopecia" derives from the Greek word Alopekia, meaning "fox mange," and broadly refers to hair loss from any hair-bearing region, most commonly the scalp. Alopecia may be congenital or acquired, localized or generalized, scarring or non-scarring, and its distribution may be focal or diffuse. Epidemiological data suggest that alopecia affects approximately 0.1-0.2% of the general population, with a lifetime risk estimated at 1.7% [4].

Telogen effluvium is defined as a diffuse, non-scarring, and reversible type of alopecia due to an abnormality in the hair cycle, occurring as a reaction pattern to various physical and mental or psychological stressors [1,2,5]. Diagnosis is primarily clinical, supported by dermoscopy - a non-invasive bedside tool capable of magnifying scalp and hair structures up to 1000×, facilitating the evaluation of follicular density, shaft morphology, and perifollicular changes [5].

Management of TE centers on patient reassurance, identification and correction of underlying triggers, and, in select cases, pharmacologic intervention with topical or oral minoxidil. Iron deficiency, particularly low serum ferritin, has emerged as a key contributor to TE in females, given iron's role in follicular proliferation and differentiation. Consequently, serum ferritin estimation serves as a critical diagnostic and therapeutic marker. This study aims to compare serum ferritin levels between female patients with telogen effluvium and matched controls.

## Materials and methods

This cross-sectional comparative study was conducted over a two-year period from June 2023 to June 2025 in the Outpatient Department of Dermatology, Venereology, and Leprosy at Sri Ramachandra Institute of Higher Education and Research, Porur, Chennai. Sample size estimation was based on findings from a prior study by Cheng et al. [6], which investigated serum ferritin levels in telogen effluvium. Assuming similar effect sizes, and applying a statistical power of 95% with a 5% level of significance, the required sample size was calculated to be 100 participants comprising 50 cases and 50 controls.

Female patients aged 18 years and above, presenting with clinically diagnosed telogen effluvium and willing to participate, were included in the study. Participants were required to consent to serum ferritin level estimation. Exclusion criteria included patients below 18 years of age, those unwilling to participate, individuals currently receiving iron therapy, and patients diagnosed with trichotillomania or other forms of alopecia, such as alopecia areata and female pattern hair loss.

Following enrollment, each participant underwent a comprehensive clinical evaluation, including detailed history taking, general and systemic examination, and trichoscopic assessment to confirm the diagnosis of telogen effluvium and exclude other causes of hair loss. Demographic and clinical parameters were recorded systematically, and standardized photographic documentation was performed for all subjects. Serum ferritin levels were measured using standard biochemical assays in both cases and controls. All samples were processed under uniform laboratory conditions to ensure consistency and reliability of results.

Data were analyzed using SPSS version 20.0 (IBM Inc., Armonk, New York). Descriptive statistics were computed to summarize continuous variables as mean and standard deviation, and categorical variables as frequencies and percentages. The independent t-test was used for continuous variables, while the Chi-squared test was employed for categorical comparisons. A p-value of less than 0.05 was considered statistically significant.

## Results

The study included 50 female patients diagnosed with telogen effluvium (TE) and 50 age-matched controls. The mean age of TE cases was 32.64 ± 10.34 years, and that of controls was 33.30 ± 10.93 years, with no statistically significant difference. Socioeconomic distribution showed that the majority of participants in both groups belonged to the lower-middle class (26 (52%) cases, 27 (54%) controls), followed by the upper-middle class. No participants in the TE group were from the lower class. Urban residence was more common in both groups (35 (70%) in cases, 32 (64%) in controls), and most participants were married (36 (72%) in cases, 41 (82%) in controls). These demographic variables did not differ significantly between groups. Menstrual irregularities were reported by 13 (26%) TE cases compared to four (8%) controls, showing a statistically significant association (p=0.017; Table 1).

**Table 1:** Demographic characteristics of telogen effluvium cases and controls *p<0.05 considered significant

Parameter	Cases (n=50)	Controls (n=50)	Test statistic	df	Effect size	p-value
Age (years)						
Mean ± SD	32.64±10.34	33.30±10.93	-0.310	98	-0.062 (Cohen's d)	0.757
Socioeconomic status, n (%)						
Lower	0 (0)	2 (4)	2.586	4	0.161 (Cramer's V)	0.629
Upper lower	6 (12)	5 (10)				
Lower middle	26 (52)	27 (54)				
Upper middle	15 (30)	12 (24)				
Upper	3 (6)	4 (8)				
Residence status, n (%)						
Urban	35 (70)	32 (64)	0.407	1	0.064 (Cramer's V)	0.523
Rural	15 (30)	18 (36)				
Marital status, n (%)						
Married	36 (72)	41 (82)	1.412	1	0.119 (Cramer's V)	0.235
Unmarried	14 (58)	9 (18)				
Menstrual history, n (%)						
Regular	37 (74)	46 (92)	5.741	1	0.240 (Cramer's V)	0.017*
Irregular	13 (26)	4 (8)				

Stress (physical and mental) was reported by 28 (56%) TE cases and 15 (30%) controls, which was also statistically significant (p=0.009). A history of dandruff was present in 31 (62%) TE cases and 18 (36%) controls, with a significant difference (p=0.009). Other lifestyle factors, such as crash dieting (three (6%) in cases vs. one (2%) in controls) and significant weight loss (six (12%) in cases vs. three (6%) in controls), were more frequent in the TE group, but they are not statistically significant. Oral contraceptive use was reported by six (12%) TE cases and two (4%) controls, while a history of polycystic ovarian syndrome (PCOS) was noted in seven (14%) TE cases and two (4%) controls. These hormonal factors showed higher prevalence in the TE group, but were not statistically significant. (Table 2)

**Table 2:** Life style and hormonal factors associated with telogen effluvium *p<0.05 considered significant OCP - oral contraceptive; PCOS - polycystic ovarian syndrome

Parameter	Cases (n=50)	Controls (n=50)	Test statistic	df	Effect size (Cramer's v)	p-value
Stress, n (%)						
Yes	28 (56)	15 (30)	6.895	1	0.263	0.009*
No	22 (44)	35 (70)				
Crash diet, n (%)						
Yes	3 (6)	1 (2)	1.042	1	0.102	0.307
No	47 (94)	49 (98)				
Significant weight loss, n (%)						
Yes	6 (12)	3 (6)	1.099	1	0.105	0.295
No	44 (88)	47 (94)				
Dandruff history, n (%)						
Yes	31 (62)	18 (36)	6.763	1	0.260	0.009*
No	19 (38)	32 (64)				
OCP usage, n (%)						
Yes	6 (12)	2 (4)	2.174	1	0.147	0.140
No	44 (88)	48 (96)				
H/o PCOS, n (%)						
Yes	7 (14)	2 (4)	3.053	1	0.175	0.081
No	43 (86)	48 (96)				

Duration of hair fall was six months or more in 31 (62%) TE cases, indicating a predominance of chronic TE, while 19 (38%) reported hair fall for less than six months. In the control group, 28 (56%) had hair fall for less than six months, and 22 (44%) for more than six months. Recession of the hairline was observed in 38 (76%) TE cases and 44 (88%) controls, with no significant difference. The average number of hairs lost per day was significantly higher in TE cases (105.60 ± 33.08) compared to controls (69.64 ± 29.69) (p<0.001). The hair pull test was positive in 31 (62%) TE cases and 17 (34%) controls, showing a statistically significant difference (p=0.005; Table 3).

**Table 3:** Hair loss patterns and clinical findings in telogen effluvium cases and controls *p<0.05 considered significant

Parameter	Cases (n=50)	Controls (n=50)	Test statistic	df	Effect size	p-value
Duration of hairfall (months), n (%)						
< 6 months	19 (38)	28 (56)	3.252	1	0.180 (Cramer's V)	0.071
≥6 months	31 (62)	22 (44)				
Recession of hair line, n (%)						
Yes	38 (76)	44 (88)	2.439	1	0.156 (Cramer's V)	0.118
no	12 (24)	6 (12)				
Number of hair loss per day						
Mean ± SD	105.60±33.08	69.64±29.69	5.720	98	1.144 (Cohen's D)	<0.001*
Hair pull test, n (%)						
Positive	31 (62)	17 (34)	7.853	1	0.280 (Cramer's V)	0.005*
Negative	19 (38)	33 (66)				

Reduced hemoglobin levels (<12 g/dL) were observed in 21 (52.5%) TE cases and nine (22%) controls (p=0.004). The mean hemoglobin level was significantly lower in TE cases (11.51 ± 1.50 g/dL) than in controls (12.59 ± 0.96 g/dL; p<0.001). Serum ferritin levels were reduced (<15 ng/mL) in 14 (28%) TE cases, while all controls had normal levels. The mean serum ferritin level was significantly lower in TE cases (24.30 ± 11.13 ng/mL) compared to controls (44.78 ± 19.89 ng/mL; p<0.001; Table 4).

**Table 4:** Haemoglobin and Serum ferritin levels in telogen effluvium cases and controls *p<0.05 considered significant

Parameter	Cases	Controls	Test statistic	df	Effect size	p-value
Hemoglobin levels (gm/dL)						
Normal (12-15), n (%)	19 (47.5)	32 (78)	8.103	1	0.316 (Cramer's V)	0.004*
Reduced (less than 12), n (%)	21 (52.5)	9 (22)				
Mean ± SD	11.51 ±1.50	12.59 ±0.96	-3.903	79	-0.867 (Cohen's D)	<0.001*
Serum ferritin levels (ng/dL)						
Normal (15-150), n (%)	36 (72)	50 (100)	16.279	1	0.403 (Cramer's V)	<0.001*
Reduced (less than 15), n (%)	14 (28)	0 (0)				
Mean ± SD	24.30 ±11.13	44.78 ±19.89	6.352	98	-1.270 (Cohen's D)	<0.001*

**Figure 1:**
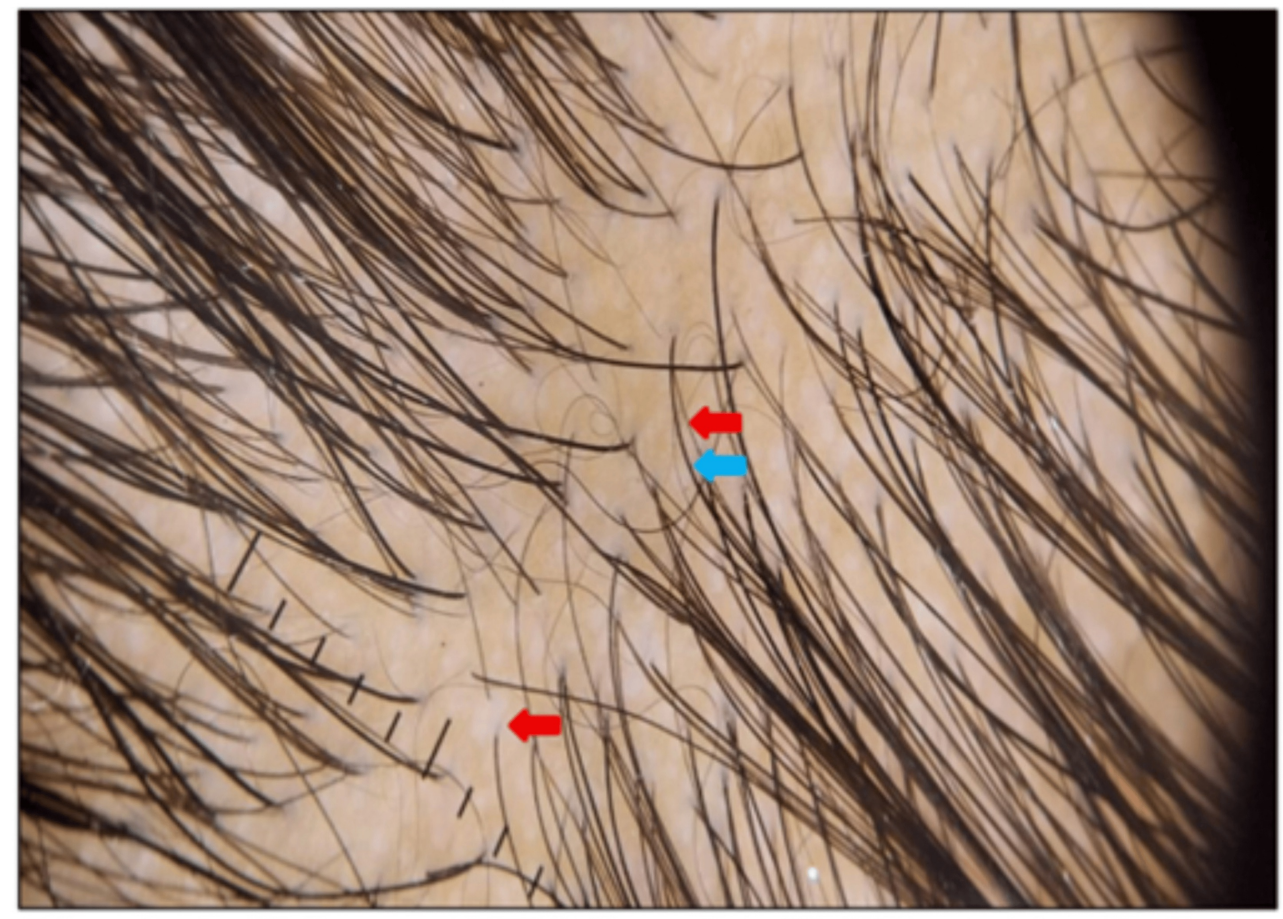
Telogen effluvium case showing follicular unit with single hair (blue arrow) and short regrowing hair (red arrow)

Trichoscopic examination revealed characteristic features consistent with telogen effluvium. The follicular unit demonstrates a single terminal hair, reflecting follicular dropout typical of telogen phase dominance. Short, regrowing hairs, suggestive of early anagen re-entry and follicular recovery (Figure 1).

## Discussion

Hair loss in females is a clinically and psychologically distressing condition with a multifactorial etiology. Common contributing factors include iron deficiency, malnutrition, helminthic infestation, endocrine dysfunction, and infectious diseases. While hemoglobin levels have traditionally been used to assess iron status, serum ferritin has emerged as a more sensitive and reliable biomarker for evaluating iron stores and their association with diffuse hair loss, particularly TE. The present study aimed to assess the correlation between serum ferritin levels in female patients with TE and matched controls.

The mean age of TE cases in this study was 32.64 ± 10.34 years, closely matching the control group (33.16 ± 10.87 years). These findings are consistent with previous studies by Ertug et al. [7] and Cheng et al. [6], who reported similar age ranges for TE cases and controls. Other studies, such as those by Ibrahem et al. [8] and Ullah et al. [9], reported slightly younger cohorts, while Fatani et al. [10] presented a wider age variability. Despite these differences, the majority of studies suggest that TE predominantly affects women between 25 and 35 years of age.

Urban residence was more common among both cases and controls, reflecting the study's tertiary care setting and broader urbanization trends. Rural representation was modest. Urban participants may have better access to healthcare and nutritional resources, potentially influencing disease recognition and management. A majority of participants were married, which may indirectly influence stress levels, dietary patterns, and health awareness. While marital status alone may not be a direct risk factor for TE, it intersects with psychosocial and lifestyle variables relevant to hair health. The study population was predominantly from lower-middle and upper-middle socioeconomic classes. Socioeconomic status affects dietary quality, access to medical care, and overall well-being - all of which are relevant to TE pathogenesis. The distribution suggests that TE affects women across economic strata, underscoring its multifactorial nature.

Acute TE, defined as hair shedding lasting less than six months, is typically self-limiting and associated with a favorable prognosis. Chronic TE, persisting beyond six months, tends to be more persistent and challenging to manage. In the present study, more than half of TE cases reported hair fall for six months or more, suggesting a predominance of chronic TE. This aligns with findings by Fatani et al. [11] who reported that 79.2% of individuals had chronic TE, while only 20.8% had acute TE. The control group showed a more balanced distribution, with a slight predominance of acute cases.

Stress was reported by more than half of the TE participants. This supports the role of psychological stress as a potential trigger or exacerbating factor in TE. However, the presence of TE in individuals without reported stress 22 (44%) indicates that TE may arise from multifactorial causes. The findings emphasize the importance of evaluating stress as part of the clinical assessment, while recognizing that it may not be universally present. 

Crash dieting, defined as consuming 800 kilocalories per day or less, was reported by three (6%) TE cases and one (2%) control. Although the prevalence was low in this cohort, it contrasts with findings by Kang et al. [12], who observed dietary factors in 47.1% of TE cases. This discrepancy may reflect differences in dietary habits, cultural norms, or assessment methods. Nutritional deprivation remains a relevant consideration in TE pathogenesis. Significant weight loss, defined as an involuntary reduction of more than 5% of body weight over 6-12 months, was reported by six (12%) TE cases and three (6%) of controls. Although the majority of participants did not report weight loss, its presence in a subset of TE cases suggests that metabolic stress and nutritional imbalance may contribute to hair shedding. Weight loss associated with illness, stress, or dietary restriction can disrupt the hair growth cycle and should be considered during evaluation.

Irregular menstrual cycles were reported by 13 (26%) TE cases, compared to four (8%) of controls. This finding is consistent with the hormonal basis of TE, where fluctuations in estrogen and progesterone can influence hair follicle dynamics. Zouboulis et al. [13] reported a higher prevalence of menstrual irregularities (41.2%) among women of childbearing age, compared to 26% in the present study. Estrogen supports hair growth, while progesterone dominance in the luteal phase may contribute to shedding. These findings underscore the importance of assessing menstrual history and considering hormonal modulation in TE management. Oral contraceptive (OCP) use was reported by six (12%) TE cases and two (4%) controls. OCPs, particularly those containing estrogen, may help regulate hormonal imbalances that contribute to hair shedding. The relatively low rate of OCP use among TE cases may reflect underutilization of hormonal therapies that could benefit women with underlying endocrine disturbances. These findings highlight the need to consider OCPs as part of a broader therapeutic strategy in selected TE cases. A history of polycystic ovarian syndrome (PCOS) was reported by seven (14%) TE cases and two (4%) controls. PCOS is associated with elevated androgen levels, which can lead to hair thinning and shedding. Although not universally present, the higher prevalence of PCOS among TE cases suggests a potential hormonal contribution to TE pathogenesis and supports the need for endocrine evaluation in affected individuals.

The hair pull test was positive in 31 (62%) of TE cases, compared to 17 (34%) of controls. These findings closely mirror those of Fatani et al. [11], who reported a 61% positivity rate. The consistency across studies supports the reliability of the hair pull test as a simple and effective diagnostic tool for detecting active hair shedding in TE.

The mean hemoglobin level in TE cases was lower compared to the controls. These findings are consistent with Fatani et al. [10] (11.56 g/dL) and Ibrahem et al. [8] (11.69 g/dL), suggesting that TE patients often present with hemoglobin levels at the lower end of the normal range. Ertug et al. [7] reported slightly higher levels (12.76 ± 1.33 g/dL), while Ullah et al. [9] observed the lowest mean (10.53 ± 1.00 g/dL), indicating variability across cohorts. These results reinforce the role of subclinical anemia in TE and the importance of hematologic screening.

In the present study, 36 (72%) of TE cases had ferritin levels within the normal range (15-150 ng/mL), with a mean of 24.30 ± 11.13 ng/mL. The remaining 14 (28%) had reduced ferritin levels (<15 ng/mL), while no control participants had ferritin below normal. The control group had a higher mean ferritin level of 44.78 ± 19.89 ng/mL. These findings are consistent with Ertug et al. [7] (17.35 ± 18.54 ng/mL), Cheng et al. [6] (24.27 ± 17.11 ng/mL), and Karakoyun et al. [14], who reported that 40.18% of TE cases had ferritin levels below 18 ng/mL. In contrast, Ullah et al. [9] observed higher ferritin levels (47.54 ± 19.85 ng/mL), and Fatani et al. [10] reported a mean of 34.30 ng/mL. The present study supports the association between reduced ferritin and TE, while acknowledging inter-study variability due to demographic, diagnostic, and regional differences. Elevated ferritin levels in some TE cases may reflect inflammation, recent supplementation, or metabolic conditions, given ferritin's role as an acute-phase reactant.

Low serum ferritin levels in telogen effluvium (TE) may result from disrupted iron metabolism due to inflammation, surgery, postpartum hemorrhage, emotional or physical stress, and dietary insufficiency-particularly in individuals following restrictive or vegan diets. Additionally, medications such as anticoagulants, anticonvulsants, and hormonal agents can alter hair follicle cycling and contribute to TE. However, ferritin levels may remain within the normal range in some TE cases, as triggers like high fever, rapid weight loss, or pharmacologic stress can induce TE independently of iron deficiency, underscoring the need for a multifactorial diagnostic approach [6,15]. Ferritin is an acute-phase reactant and may be elevated in inflammatory conditions independent of iron status. The absence of erythrocyte sedimentation rate (ESR) and C-reactive protein (CRP) limits the ability to exclude occult inflammation completely. However, all participants were clinically evaluated and did not exhibit features suggestive of systemic inflammatory disease.

Thus, this study reinforces the association between reduced serum ferritin levels and telogen effluvium (TE) in women, particularly within a tertiary care urban population. While ferritin may not be universally low in all TE cases, its role as a sensitive indicator of iron deficiency highlights the importance of routine nutritional screening in patients presenting with diffuse hair loss. Integrating ferritin assessment into dermatologic and primary care protocols may facilitate earlier diagnosis, targeted intervention, and improved patient outcomes.

This study was limited by its small sample size, single‑center design, and relatively short duration. Self‑reported data on stress, diet, and menstrual history may be subject to recall bias. Serum ferritin levels can also be affected by factors such as inflammation or recent supplementation, which were not fully controlled. In addition, the treatment status of dandruff was not considered, which may influence hair shedding. Finally, variability in dieting patterns and recall bias prevented precise documentation of crash‑diet duration for all participants.

## Conclusions

Serum ferritin has emerged as a novel tool for estimating the prevalence of iron deficiency, which is associated with diffuse hair loss, specifically telogen effluvium. The present study, conducted at a tertiary care hospital in an urban setting, may represent a reasonable sketch of this background. Further large population-based assessments will go a long way in helping us to get further insights into this entity, highlighting the primacy of diet and nutrition in healthy hair, so as to ensure a good quality of life in the future.
